# Meeting the need for surgery

**DOI:** 10.2471/BLT.16.020316

**Published:** 2016-03-01

## Abstract

Surgical provision falls far short of what is needed in developing countries, but recent initiatives aim to correct this deficit. Carolyn Mahoney and Fiona Fleck report.

Malawi’s health system relies heavily on clinical officers. In eastern and southern Africa countries, these professionals are qualified to provide specialized and general medical services that in other countries would be the preserve of physicians.

Clinical officers like Calistus Chiumia can diagnose and treat disease and injury, perform routine medical and surgical procedures and, often, run district hospitals because there are so few physicians in his country.

“There is a great need for surgery in my community. Our central hospitals are very far from our district, but they are still congested with patients because so many of them need surgery,” says Chiumia, who works in Mangochi District Hospital in southern Malawi.

“If the surgical expertise were available on a district level, it would be different,” Chiumia says.

Like so many other clinical officers in Africa, Chiumia was initially trained in pregnancy-related surgical procedures such as caesarean sections. He also acquired other surgical skills on the job at the district hospital.

Four years ago, he started his specialized training in surgery with a group of surgeons from Ireland under the European Union-funded Clinical Officer Surgical Training in Africa (COST) initiative.

“There is a great need for surgery in my community.”

Calistus Chiumia

This year, Chiumia graduates with his bachelor of science degree in general surgery and will be qualified to do additional surgical procedures, such as hydrocele or hernia repairs. He is also trained to recognize cases that need referral to the central hospital for surgery.

For Dr Stephen Ogendo, who recently stepped down as president of the College of Surgeons of East, Central and Southern Africa (COSECSA), sub-Saharan Africa countries will not meet the need for surgery without training more clinical officers as surgeons.

“Half of the countries in sub-Saharan Africa already use non-physician surgeons,” says Ogendo, a professor of surgery at Nairobi University in Kenya.

“They provide basic, low-cost essential surgical interventions in district hospitals. For example, in Malawi, Mozambique and the United Republic of Tanzania, 85–90% of caesarean sections, obstetric hysterectomies and laparotomies for ectopic pregnancy are performed by non-physician surgeons,” Ogendo says.

“It costs less and takes less time to train clinical officers as surgeons, but still there is still a high unmet need for surgery in these countries,” he says.

This unmet need has long been recognized.

In 1980, two years after the Alma-Ata “Health for All” Declaration, former Director-General of the World Health Organization (WHO), Dr Halfdan Mahler, spoke of the central role of surgery in primary care.

“The distribution of surgical resources throughout the world must come under scrutiny in the same way as any other intellectual, scientific, technical, social or economic commodity. The era of only the best for the few and nothing for the many is drawing to a close,” Mahler told the World Congress of the International College of Surgeons in Mexico in that year, making access to surgery a social justice issue.

Writing in the *World Journal of Surgery* in 2008, Dr Paul Farmer and Dr Jim Kim made an impassioned plea to strengthen surgical care in these countries, calling surgery in Africa “the neglected stepchild of global health”.

But today little has changed in terms of access to surgery in developing countries.

The volume of surgery increased globally between 2004 and 2012, but wide disparities persist between rich and poor countries, according to a study published in this issue of the *Bulletin of the World Health Organization*.

An estimated 288.2 million people in the poorest 48 countries are in need of surgical care, according to one of a series of studies published for the Lancet Commission on Global Surgery in 2015.

The Lancet Commission estimates that five billion people lack access to safe, timely and affordable surgery. According to *Disease Control Priorities in Developing Countries*, third edition, an estimated 1.5 million deaths per year could be prevented by making basic surgical procedures accessible.

Despite considerable progress in global health, the development of essential surgical services in low- and middle-income countries “has stagnated or even regressed” over the last 25 years, members of the Lancet Commission write.

“Case-fatality rates are high for common, easily treatable conditions including appendicitis, hernia, fractures, obstructed labour, congenital anomalies, and breast and cervical cancer,” they continue.

In May 2015, the World Health Assembly passed a unanimous resolution calling for “strengthening of emergency and essential surgical care including the provision of anaesthesia as a component of universal health coverage”.

“These three developments – the *Disease Control Priorities *report, the Lancet Commission and the resolution – have really produced a groundswell of support and enthusiasm for global surgery,” says Dr Walter Johnson, who coordinates WHO’s Emergency and Essential Surgical Care Programme.

The College of Surgeons of East, Central and Southern Africa has been building surgical capacity in 10 countries (Burundi, Ethiopia, Kenya, Malawi, Mozambique, Rwanda, the United Republic of Tanzania, Uganda, Zambia and Zimbabwe) for many years.

A study in the *East and Central African Journal of Surgery* highlighted the barriers to providing adequate surgical care in sub-Saharan African countries, citing limited government health-care budgets and that donors tended to focus on infectious diseases.

But the shift in the burden of disease to noncommunicable diseases, the authors argue, presents an opportunity to raise more funds for surgical capacity, since “cancer, trauma and aspects of patient safety … are increasingly becoming of public health concern and do involve significant amounts of surgical input”.

Good training is essential, considering that many clinical officers, midwives and other mid-level health workers in developing countries are already performing essential surgery but are not always fully trained or qualified in surgery.

“We’re reaching out to the people who know the most about training, like COSECSA, the American College of Surgeons, many of the royal colleges of surgeons, all partnering to help with designing minimum standard curricula for training for mid-level health workers, and then how to credential them and denote competency,” Johnson says,

Through the efforts of COSECSA and other groups, such as the COST initiative that trains clinical officers in Malawi and Zambia, progress has been made in standardizing training for surgeons in these countries.

WHO has developed guidelines and checklists for surgical care related to injuries, congenital abnormalities, cancer, infectious disease and childbirth and these are available on the WHO website, including *Surgical care at the district hospital* and the *Integrated management of emergency and essential surgical care* *toolkit*, which includes resources to improve surgical care programmes.

In 2005, WHO launched the Global Initiative for Emergency and Essential Surgical Care to support low- and middle-income countries in their efforts to meet the need for emergency, anaesthesia, and surgical care in primary health-care facilities.

Three years later, Safe Surgery Saves Lives was established to deﬁne core safety standards for adoption by WHO’s Member States.

“My task now is to start developing a roadmap towards implementing the World Health Assembly resolution. I’m trying to bring surgeons, anaesthetists and public health experts all to the table to talk about the best ways to do this,” Johnson says.

The challenges go beyond the need for training and credentialing.

District hospitals often lack reliable supplies of water, oxygen, electricity and anaesthetics or even surgical gloves, making even the most basic surgery challenging if not impossible.

“It is very difficult to work with very few resources and, sometimes, it lowers the morale because you cannot to do the things that you would have otherwise done,” says Chiumia.

The lack of basic supplies extends to medicines routinely available in developed countries: “About 80% of all the narcotics used in the world are consumed in six countries – all of them high-income countries,” Johnson says.

“For a big abdominal procedure in sub-Saharan Africa – a colectomy or hysterectomy, for example – you get an over-the-counter painkiller. So access to anaesthesia and post-anaesthesia care is also part of the problem,” he says.

Those, like Johnson, who are engaged in trying to change the situation must battle preconceptions that surgery is too costly or complex for low-income countries.

“We need to see surgery as an investment, not a cost. It’s an important distinction.”

Walter Johnson

“Most people assume that surgery is very expensive. But it’s been shown for a number of years that it’s actually quite cost effective,” Johnson says.

“To bring the 88 lowest-income countries up to the standard-of-care of middle-income countries would cost about US$ 420 billion over 15 years, which seems like a lot,” Johnson says.

“But if you don’t do that, the total cost in disability and lost productivity would be more than US$ 12 trillion over the same time period – so it’s a very good investment,” he says.

“We need to see surgery as an investment, not a cost. It’s an important distinction.”

**Figure:**
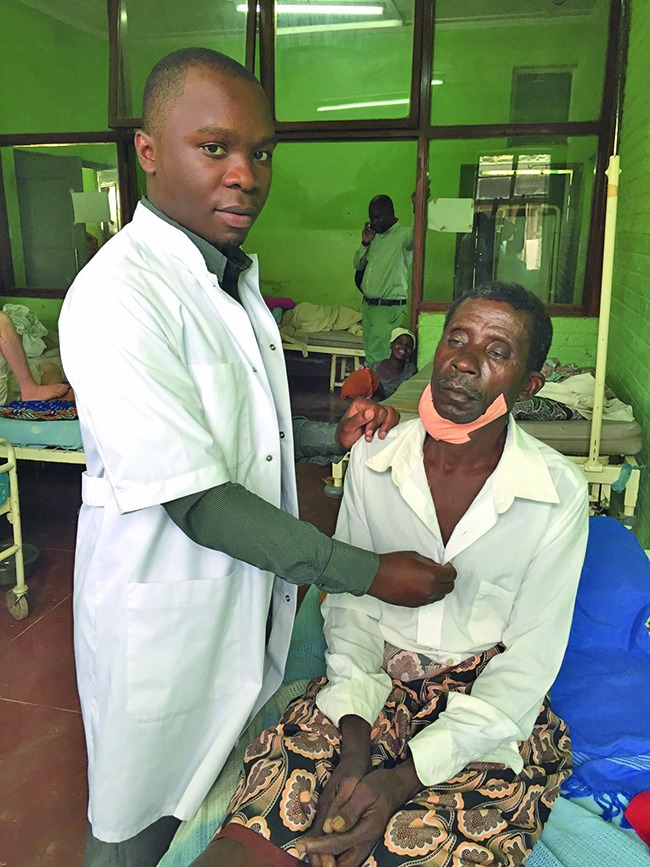
Clinical Officer Calistus Chiumia examines a patient at Mangochi District Hospital in southern Malawi.

Courtesy of Clinical Officer Surgical Training in Africa/Jakub Gajewski

**Figure:**
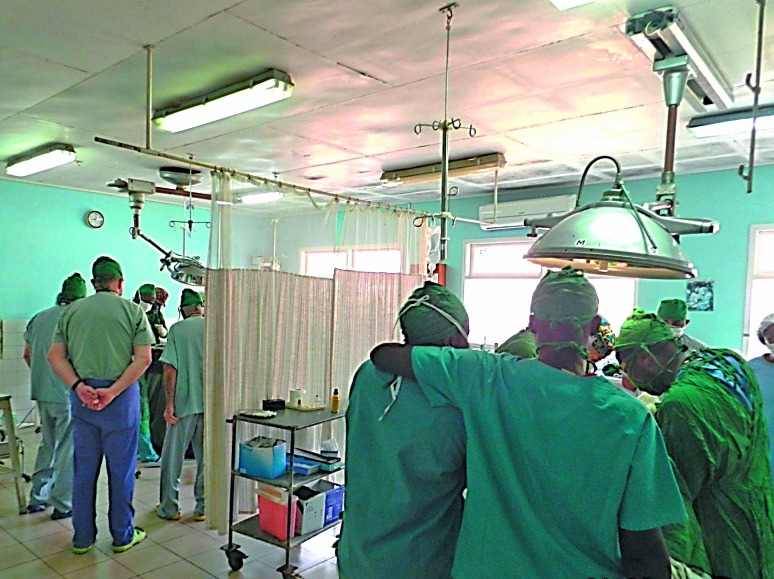
Two operations being done simultaneously in an operating theatre in a rural hospital in Cameroon. The hospital runs a training programme for local surgeons.

Walter Johnson

